# Childhood Renal Tumor: A Report from a Chinese Children's Cancer Group

**DOI:** 10.1155/2014/894341

**Published:** 2014-07-24

**Authors:** Jiaoyang Cai, Ci Pan, Qin Lu, Jie Yan, Xiuli Ju, Futian Ma, Yiping Zhu, Qiuling Liu, Lirong Sun, Lian Jiang, Lizhi Cao, Fu Li, Zhigang Liu, Lijing Qiao, Dongsheng Huang, Xin Tian, Jingyan Tang

**Affiliations:** ^1^Key Laboratory of Pediatric Hematology & Oncology Ministry of Health, Department of Pediatric Hematology and Oncology, Shanghai Children's Medical Center, Shanghai Jiao Tong University School of Medicine (SJTU-SM), Shanghai, China; ^2^Nanjing Children's Hospital Affiliated to Nanjing Medical University, Nanjing, Jiangsu, China; ^3^Tumor Hospital Affiliated to Tianjin Medicine University, Tianjin, China; ^4^Qilu Hospital Affiliated to Shandong University, Qingdao, Shandong, China; ^5^The Second Affiliated Hospital of Hebei Medical University, Shijiazhuang, Hebei, China; ^6^West China Second University Hospital of Sichuan University, Chengdu, Sichuan, China; ^7^Chinese People's Armed Police Force General Hospital, Beijing, China; ^8^The Affiliated Hospital of Qingdao University, Qingdao, Shandong, China; ^9^The Fourth Hospital Affiliated to Hebei Medical University, Shijiazhuang, Hebei, China; ^10^Xiangya Hospital Affiliated to Central South University, Changsha, Hunan, China; ^11^Qilu Children's Hospital Affiliated to Shandong University, Qingdao, Shandong, China; ^12^Xi'an Jiaotong University First Affiliated Hospital, Xi'an, Shanxi, China; ^13^TianJin Children's Hospital, Tianjin, China; ^14^Beijing Tongren Hospital, Beijing, China; ^15^Kunming Children's Hospital, Kunming, Yunnan, China

## Abstract

Here we investigated the establishment of multicenter cooperative treatment groups in China, as well as radiotherapy compliance and effectiveness among children with renal tumors. Medical records were reviewed for 316 children with renal tumors diagnosed by a multicenter cooperative group from 14 hospitals in China from 1998 to 2012. Median patient age was 29.5 months (range, 2–173 months old), and male-to-female ratio was 1.4 : 1. After a median follow-up of 22 months (range, 1–177 months), five-year event-free survival rates were 72% overall; 76.1% for favorable histology (251 cases); 59% for unfavorable histology (27 cases); and 91%, 75%, 71%, 53%, and 48.5%, respectively for Stages I, II, III, IV, and V. Following standardized criteria, radiation therapy was indicated for 153 patients, among whom five-year event-free survival was 72.8% for the 95 who received radiation and 24% for the 58 patients who did not. Our results are reasonable but can be further improved and show the feasibility of a multicenter cooperative group model for childhood renal tumor treatment in China. Radiation therapy is important for stage III and IV patients but remains difficult to implement in some parts of China. Government management departments and medical professionals must pay attention to this situation. This clinical trial is registered with ChiCTR-PRCH-14004372.

## 1. Introduction

Accounting for 5-6% of all malignant childhood tumors, renal tumors are among the most common malignant solid tumors in children, with Wilms' tumor being the most common childhood renal malignancy [1]. The introduction of radiation therapy (RT) for Wilms' tumor in the 1940s increased the cure rate to nearly 50%, and addition of single-agent chemotherapy in the 1950s further improved the 2-year survival rate to 60–80% [2, 3]. The outcome for children with renal tumors has also improved with the refinement of multimodal therapy—which includes surgery, chemotherapy, and sometimes RT—such that overall survival rates now approach 90% in developed countries [4, 5].

Over recent decades, treatment planning by collaborative groups and multidisciplinary teams has contributed immensely to increasing survival in developed countries; however, such models are not yet widely implemented in developing countries. Starting in 1998, at Shanghai Children's Medical Center in China, we have followed this effective working model for treatment of childhood cancer, including renal tumors. We organized a multidisciplinary team of oncologists, surgeons, pathologists, and subspecialized radiologists and established a tumor board for coordinating diagnosis, treatment evaluation, and patient transfer among these specialists. To acquire large sample data, we also set up a multicenter cooperative group from 15 pediatric centers that have treated childhood renal tumors using uniform treatment guidelines adapted to local circumstances in China since 1998. The present study aimed to evaluate whether RT treatment had a significant impact on the outcome for childhood renal tumor patients admitted to a multicenter cooperative group and treated under a multidisciplinary teamwork model.

## 2. Materials and Methods 

We performed a retrospective review of patients with a pathologically proven diagnosis of renal tumor—including Wilms' tumor, clear cell sarcoma, rhabdoid tumor, and other renal tumors—from a multicenter cooperative group of 14 hospitals in China from December 1998 through September 2012. One of the originally selected 15 hospitals was rejected because it did not conform to the requirements. This study was approved by the Institutional Review Board of Shanghai Children's Medical Center. Diagnosis and treatment were decided by a multidisciplinary team that included oncologists, surgeons, pathologists, and subspecialized radiologists. The medical records of patients with renal tumors were reviewed for age at diagnosis, sex, mode of presentation, involved kidney, preoperative treatment, type of surgery, stage, postoperative treatment modalities, follow-up period, and outcome (including complications, tumor recurrence, and survival).

An unfavorable histology is an anaplastic one detectable by the presence of gigantic polypoid nuclei within the tumor sample [6]. The criteria distinguishing focal from diffuse anaplasia on previous NWTSG protocols were modified. The original definition of focal anaplasia was based on the amount of anaplasia present. The new definition emphasizes the distribution of anaplasia [7]. The lack of anaplasia was considered a favorable histological feature. Clear-cell sarcoma of the kidney and malignant rhabdoid tumor of the kidney are now considered distinct tumor types and were separately evaluated in our study. In all patients, clinical staging was determined according to the criteria of the Third and Fourth National Wilms' Tumor Study Group (NWTSG), based exclusively on the anatomic extent of the tumor, without considering genetic, biological, or molecular markers. Histological classification was also as defined by the NWTSG study.

The regime of systemic chemotherapy was worked out according to the NWTSG protocol. Patients with Stage I-II favorable histology and with Stage I focal anaplastic histology received WT (1) (Dactinomycin and Vincristine) for 19 weeks. Patients with Stage III-IV favorable histology, with Stage II-III focal anaplastic and with Stage I diffuse anaplastic received WT (2) (Doxorubicin, Dactinomycin, and Vincristine) for 25 weeks. Patients with Stage II-III diffuse anaplastic, with Stage I–III clear cell sarcoma, and with Stage IV focal anaplastic received WT (3) (Cyclophosphamide, Doxorubicin, Vincristine, and Etoposide) for 25 weeks. Patients with Stage I–IV rhabdoid tumor, with Stage IV diffuse anaplastic, and clear cell sarcoma received WT (4) (Carboplatin, Cyclophosphamide, Doxorubicin, Vincristine, and Etoposide) for 27 weeks. Patients with Stage IV or unresectable Stage III tumor received WT (5) (Ifosfamide, Vincristine, and Etoposide) for six weeks and were reassessed for feasibility of surgical management then switched to the regimen after surgery depending on the original staging. Various drug doses were showed in supplement Table 1 available online at http://dx.doi.org/10.1155/2014/894341.

Most patients with unilateral renal tumors were treated surgically, followed by postoperative chemotherapy with or without RT. The exact protocol was determined according to the NWTS protocols and depended on the patient's age and the stage of the tumor. Preoperative chemotherapy was administered to patients with bilateral Wilms' tumor (BWT) or with a tumor that could not be removed completely at the first presentation.

For patients whose primary tumors were initially resected, RT was started within 10 days after operation when indicated.  Table 1 shows the indications for radiation therapy. For patients younger than 12 months of age, RT was omitted or delayed until the child reached 12 months old. For patients with liver and/or lung metastatic diseases, the decision of whether to administer metastatic site RT was made based on discussion between the physician, radiologist, and parents.

### 2.1. Statistical Methods

Event-free survival (EFS) was defined as the time from study entry to the first occurrence of progression, relapse, and death from any cause or loss to follow-up. Survival was defined as the time from study entry to death from any cause. Patients without events were censored at the time of their last follow-up. The collected data were analyzed using SPSS software, version 13.0. Survival rates were assessed using the Kaplan-Meier method.

## 3. Results

During the study period, 316 children diagnosed as having renal tumors were admitted to a multicenter cooperative group in China. Of these patients, 186 were male and 130 were female (M/F = 1.4). The median age at the time of diagnosis was 29.5 months (range, 2–173 months); 248 (78.5%) were between 0–4 years old, of which 66 were <1 year old, 70 were 1-2 years old, 66 were 2-3 years old, and 46 were 3-4 years of age. Favorable histology (FH) was diagnosed in 251 patients (79.4%), unfavorable histology (UFH) in 27 patients (8.5%), clear cell sarcoma in 24 patients (7.6%), rhabdoid tumor in 11 patients (3.5%), and undifferentiated renal tumor in 3 patients (0.9%). Tumor stage was determined at initial exploration, with 86 patients (27.2%) designated Stage I, 98 (31.0%) Stage II, 80 (25.3%) Stage III, 41 (13.0%) Stage IV, and 11 (3.5%) Stage V.

**Figure 1:**
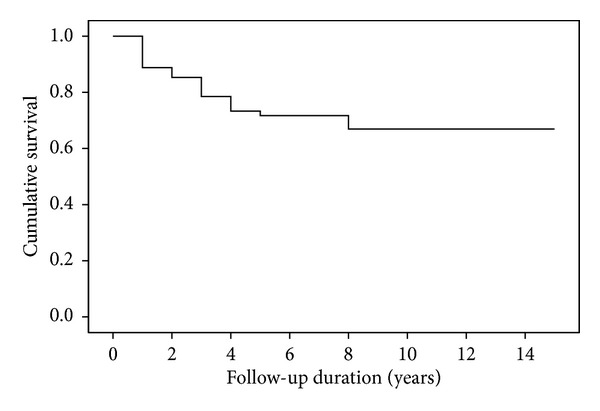
Kaplan-Meier estimations of event-free survival for 316 renal tumor patients.

**Figure 2:**
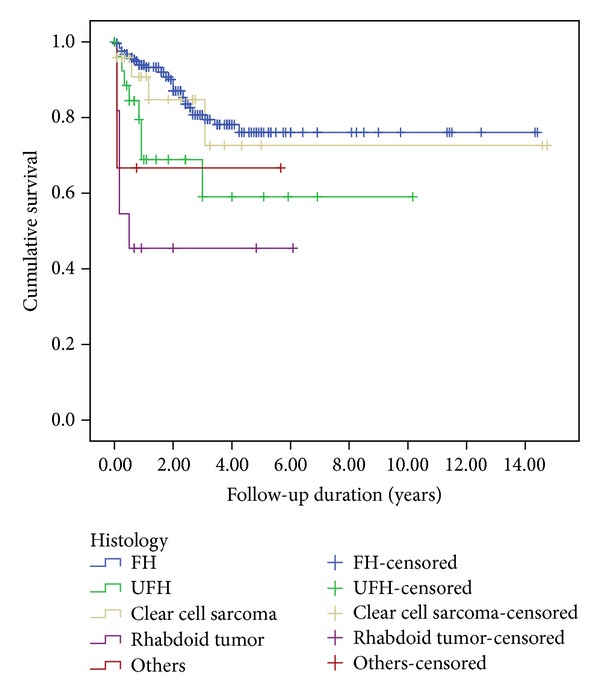
Kaplan-Meier estimations of event-free survival for various groups of renal tumor patients. FH, favorable histology; UFH, unfavorable histology.

**Figure 3:**
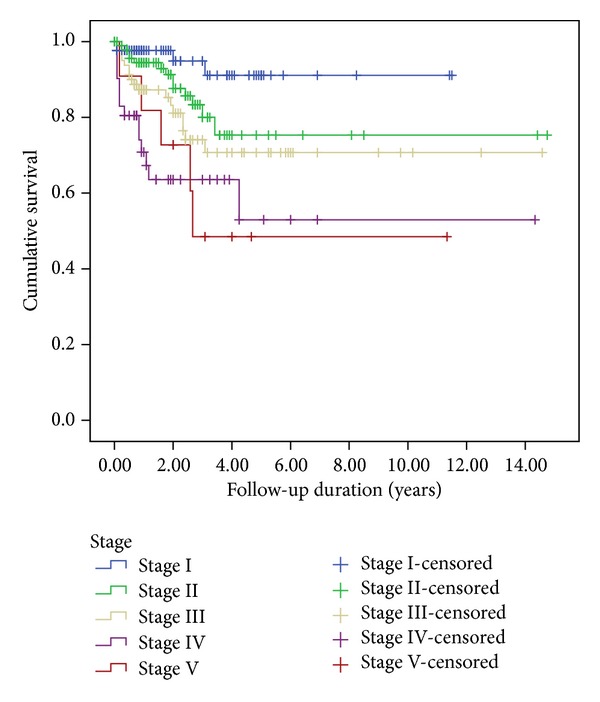
Kaplan-Meier estimations of event-free survival for 316 renal tumor patients according to stage.

The median follow-up of all patients was 22 months (range, 3–177 months). The five-year EFS rates were 72% for all patients (Figure 1), 79% for FH (55 cases), 59% for UFH (27 cases), 73% for clear cell sarcoma (24 cases), and 46% for rhabdoid tumor (11 cases). Survival rates significantly differed between groups with FH, UFH, clear cell sarcomas, and rhabdoid tumors (*P* = 0.000; Figure 2). The five-year EFS rates were 91.1% for Stage I, 75.3% for Stage II, 70.7% for Stage III, 53% for Stage IV, and 48.5% for Stage V (Figure 3).

**Figure 4:**
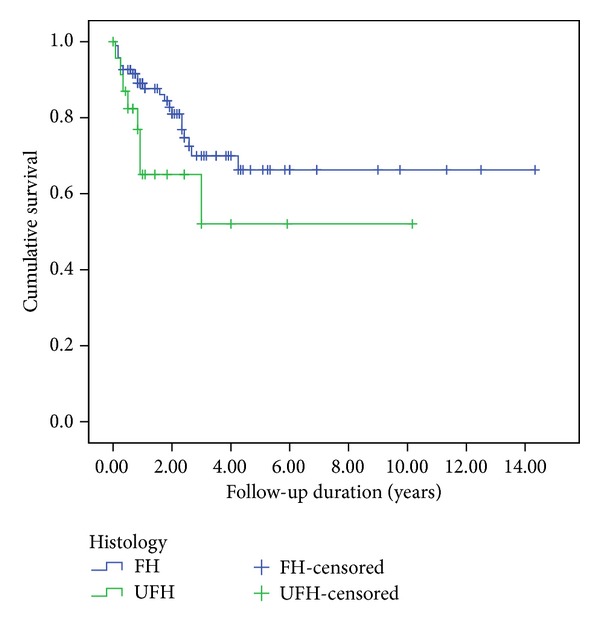
Kaplan-Meier estimations of EFS for 95 FH and 24 UFH patients who were administered RT.

The protocol indicated that RT should have been administered to 153 children following the initial surgery. Of these 153 patients, 95 were diagnosed with FH, 24 UFH, 20 clear cell sarcoma, 11 rhabdoid tumor, and 3 other renal tumor. The stages distribution among these 153 patients were 3 Stage I, 17 Stage II, 81 Stage III, 41 Stage IV, and 11 Stage V (Table 2). The five-year EFS rates among these 153 cases were 66.3% for FH and 52% for UFH patients. (*P* = 0.096; Figure 4).

Tables 3 and 4 report the comparisons of five-year EFS rates by histology and stage for the 153 patients with RT or without RT.

**Figure 5:**
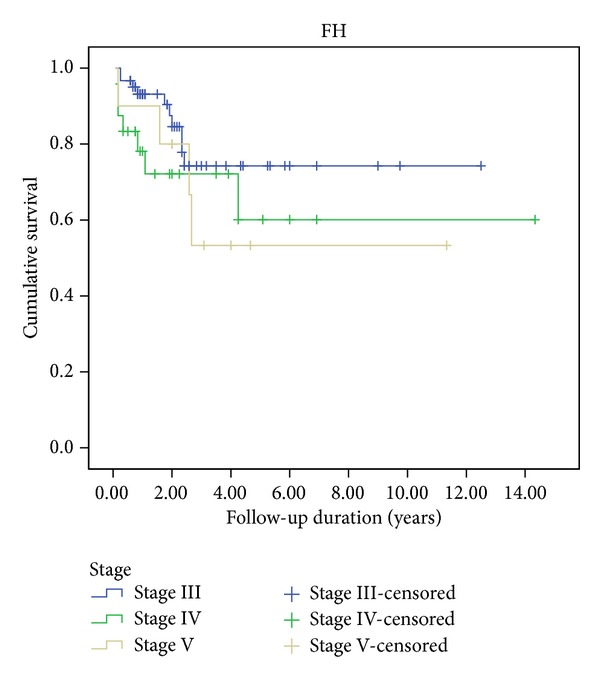
Kaplan-Meier estimations of EFS for 95 FH patients who were administered to RT by Stage.

Among the 95 FH patients for which RT was indicated, the five-year EFS rates were 74.3%, 60.1%, and 53.3% for Stages III, IV, and V, respectively (Figure 5).

**Figure 6:**
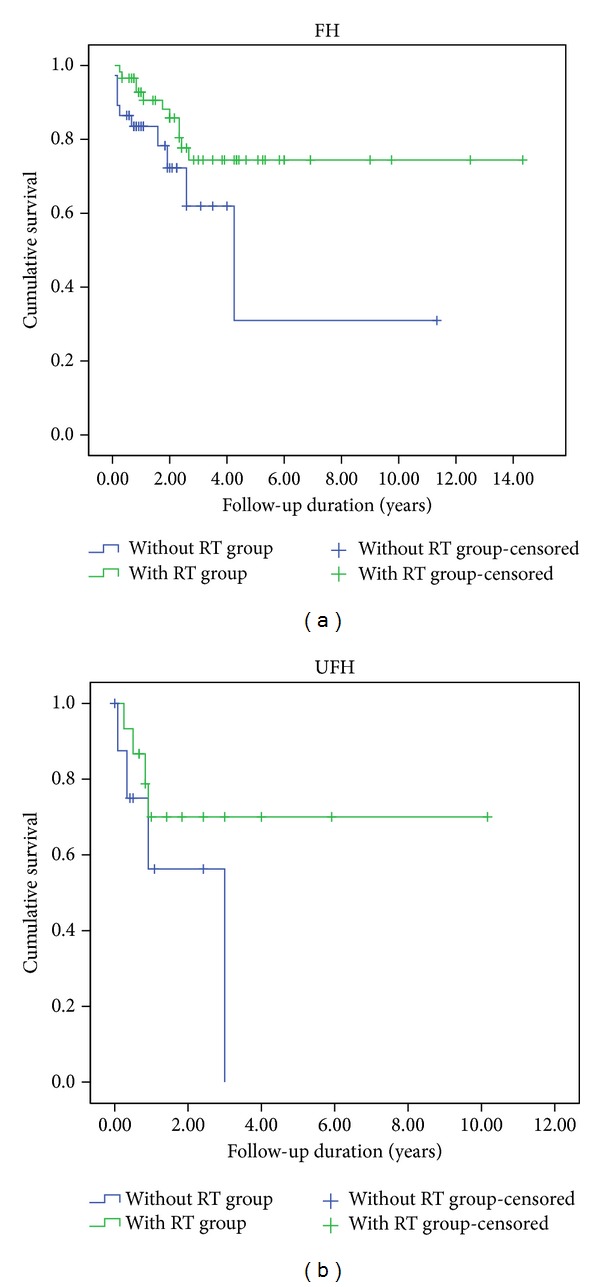
Kaplan-Meier estimations of EFS for renal tumor patients (FH and UFH) who were indicated to receive radiotherapy and who did or did not actually undergo radiotherapy.

Of the 95 FH patients indicated to receive RT based on the protocol, 58 (61%) actually underwent RT. The remaining 37 patients (39%) did not receive RT due to lack of local radiation facilities or child-specialized radiotherapy experts or because the patients' parents failed to comply. Among patients who should have received RT, five-year EFS rates significantly differed between cases with RT (74.4%) and without RT (31%) (*P* = 0.043; Figure 6). Of the 24 UFH patients indicated to receive RT based on the protocol, 15 (62.5%) actually underwent RT. The remaining 9 patients (37.5%) did not receive RT. Among patients who should have received RT, five-year EFS rates significantly differed between cases with RT (70%) and without RT (31%) (*P* = 0.01; Figure 6).

Three patients (3.1%) had hepatic venoocclusive disease (VOD) marked by hepatomegaly, ascites, and increased bilirubin at 33–63 days after RT—which was recovered within 15 days in each case. Among the 25 patients with liver and/or lung metastatic diseases, only two received metastatic site RT (both in the lung), based on a decision made by the physician, radiologist, and parents. Among these patients, one of the 2 who received lung irradiation relapsed, while 7 of the 23 who did not receive lung irradiation relapsed. No cardiac toxicity, renal failure, lung toxicity, or toxic deaths occurred in our study.

## 4. Discussion

Compared to developed countries, China has lower rates of long-term event-free survival in cases of childhood renal tumors. Developing countries face several specific challenges when treating children with renal tumors. Children will often present late with advanced disease, and failure to complete treatment is a common cause of treatment failure. Furthermore, surgery, chemotherapy, and radiotherapy are often received in different hospitals, with no communication between different specialists. These challenges must be taken into account when developing treatment guidelines adapted to local conditions. The present multicenter cooperative group study was developed to improve these situations.

The percentage of Stage I tumors in our present study (27.2%) was lower than that reported in NWTS-5 (35%). This difference may be due to the delay in the presentation of some of our patients. Additionally, the rate of favorable histology in our study was 79.4%, compared with 92.2% in NWTS-5 [8], which may account for the poor prognosis in our study compared to in NWTS-5. According to NWTS-5 results, surgery alone may be adequate treatment for a limited group of children who are younger than 2 years of age at diagnosis and have Stage I Wilms' tumors with favorable histology that weigh less than 550 grams [9]. Accordingly, we could adjust our protocol for Stage I patients to avoid unnecessary chemotherapy. In our study, 3.5% of patients were Stage V; these patients showed very poor prognosis with an overall five-year survival rate of 48.5%. Our overall survival rates were lower than those of SIOP (overall 10-year survival rate, 69%) [10, 11].

Based on stage and histology, RT should have been administered to 153 children in our present study. Of these 153 patients, 37.9% did not receive RT for various reasons, including the lack of radiotherapy facilities in most of the children's hospital. Other reasons included a lack of children-specialized radiotherapy experts, and patients' parents' failure to comply. Among the patients indicated to receive RT, EFS rates significantly differed between those who did and did not actually undergo RT, according to different histological features and stages. This finding shows that appropriate administration of RT plays a very important role in this protocol. Late effects of high radiation doses can lead to growth retardation, function impairment, carcinogenesis, and neurocognitive deficits. In our study, three patients experienced VOD between 33 to 63 days after radiation therapy, and no other side effects of radiation therapy were reported.

In the NWTS-4, the two-year relapse-free survival rate for patients with Stage IV disease was 81% [12]. In the United Kingdom Wilms' Tumor Study (UKWS 2/3), the four-year EFS rates for Stages III, IV, and V were 82%, 70%, and 70%, respectively [13, 14]. Compared to these previous studies in developed countries, our presently reported survival rate was poor. However, if we restrict our analysis to the patients who strictly followed the protocol and accepted local RT, our results are close to those of these previous studies.

The first Wilms' tumor study by the United Kingdom Children's Cancer Study Group reported a survival rate of only 65% in patients with lung metastases who did not receive radiation therapy [15]. Of the 25 patients in our study with pulmonary metastases, 2 received lung irradiation, of which 1 relapsed. Of the 23 who were not radiated, 7 relapsed (4 cases in lung and 4 cases in abdomen). Our present findings raise questions about the role of lung irradiation.

## 5. Conclusion

A multicenter cooperative group model for childhood renal tumor treatment is feasible in China. The present results are reasonable but can be further improved. Radiation therapy is important for stage III and IV patients, and it should be administered when indicated. In some parts of China, it remains difficult to implement radiation therapy, and government management departments and medical professionals must pay attention to this situation.

## Supplementary Material

Supplementary Table 1. Treatment plan: Regime WT(1)-(5). Patients with Stage I-II favorable histology or with Stage I focal anaplastic histology received WT-1. Patients with Stage III-IV favorable histology, with Stage II-III focal anaplastic histology, or with Stage I diffuse anaplastic histology received WT-2. Patients with Stage II-III diffuse anaplastic histology, with Stage I-III clear cell sarcoma, or with Stage IV focal anaplastic histology received WT-3. Patients with Stage I-IV rhabdoid tumor or with Stage IV diffuse anaplastic histology and clear cell sarcoma received WT-4. Patients with Stage IV or unresectable Stage III tumor received WT-5.

## Figures and Tables

**Table 1:** Radiation dose and volume by tumor stage or clinical presentation.

Stage III-IV favorable histology	(1) Whole abdomen irradiation (WAI) 10.8 Gy in six 180 cGy fractions. Supplemental doses of 1080 cGy are given to patients with residual tumor.
	(2) Metastatic sites: liver irradiation 19.8 Gy, lesser volumes may receive 540 to 1080 cGy, 3060 cGy doses should not be given to more than 75% of the liver volume; whole lung irradiation 12 Gy followed by an additional 750 cGy; lymph node irradiation 19.8 Gy followed by a local boost of 5.4–10.8 Gy; whole brain irradiation 30.6 Gy; bone irradiation 30.6 Gy.

Stage II–IV anaplasia	(1) Patients will receive supplemental “boost” irradiation 19.8 Gy. Whole abdomen irradiation (WAI) 19.8 Gy followed by a flank boost 9 Gy are given to patients with stage III-IV anaplasia.
	(2) Metastatic sites: same as stage IV favorable histology.

Stage II–IV clear cell sarcoma	(1) Supplemental irradiation 10.8 Gy are given to patients with stage II clear cell sarcoma. Whole abdomen irradiation (WAI) 10.8 Gy followed by a local boost. Metastatic sites are given to patients with stage III clear cell sarcoma.
	(2) Metastatic sites: same as stage IV favorable histology.

Stage I–IV rhabdoid tumor	(1) Whole abdomen irradiation (WAI) 19.8 Gy followed by a local boost. Patients 12 months or younger will have their total dose reduced to 10.8 Gy.
	(2) Metastatic sites: same as stage IV favorable histology.

**Table 2:** Patient characteristics of the 153 patients who were administered RT.

Stage	With RT		Without RT	
	Number of cases	Histology	Number of cases	Histology
I	0	—	3	3 rhabdoid
II	10	4 UFH, 5 clear cell, and 1 rhabdoid	7	4 UFH, 3 clear cell
III	56	41 FH, 5 UFH, 4 clear cell, 5 rhabdoid, and 1 undifferentiated	25	20 FH, 3 UFH, and 2 clear cell
IV	26	14 FH, 6 UFH, 5 clear cell, and 1 undifferentiated	15	10 FH, 1 UFH, 1 clear cell, 2 rhabdoid, and 1 undifferentiated
V	3	3 FH	8	7 FH and 1 UFH

**Table 3:** The five-year EFS rates among 153 patients indicated to receive radiotherapy, according to whether they actually underwent radiotherapy and histology.

Histology	Five-year EFS rates (number of cases)	
	With RT	Without RT
FH	74% (58)	31% (37)
UFH	70% (15)	0% (9)
Clear cell	76% (14)	56% (6)
Rhabdoid	50% (6)	40% (5)
Undifferentiated	100% (2)	0% (1)

Total	72.8%	24%

**Table 4:** The five-year EFS rates for 153 patients indicated to receive radiotherapy, according to whether they actually underwent radiotherapy and stage.

Stage	Five-year EFS rates (number of cases)	
	With RT	Without RT
I	—∗ (0)	66.7% (3)
II	90% (10)	85.7% (7)
III	73.1% (56)	63.3% (25)
IV	76.1% (26)	0% (15)
V	50% (3)	46.9% (8)

Total	72.8%	24%

*Only stage I rhabdoid tumor patients indicated to receive RT based on the protocol and none of them actually underwent radiotherapy.
