# Supplementary material for: Childhood Renal Tumor: A Report from a Chinese Children's Cancer Group
Source: Biomed Res Int. 2014 Jul 24;2014:894341. doi: 10.1155/2014/894341 (PMC4131417; doi:10.1155/2014/894341)
Supplement: Supplementary file 1 — Supplementary Table 1. Treatment plan: Regime WT(1)-(5). Patients with Stage I-II favorable histology or with Stage I focal anaplastic histology received WT-1. Patients with Stage III-IV favorable histology, with Stage II-III focal anaplastic histology, or with Stage I diffuse anaplastic histology received WT-2. Patients with Stage II-III diffuse anaplastic histology, with Stage I-III clear cell sarcoma, or with Stage IV focal anaplastic histology received WT-3. Patients with Stage I-IV rhabdoid tumor or with Stage IV diffuse anaplastic histology and clear cell sarcoma received WT-4. Patients with Stage IV or unresectable Stage III tumor received WT-5. [file 894341.f1.docx]

SUPPLEMENT table 1. **Treatment plan: Regime WT(1)-(5).**

**WT (1) week**

1 2 3 4 5 6 7 8 9 10 11 12 13 14 15 16 17 18 19

**DACT DACT DACT DACT DACT DACT DACT**

**VCR VCR VCR VCR VCR VCR VCR VCR VCR VCR VCR* VCR* VCR***

DACT (Dactinomycin) IV on Day 1. 0.023 mg/kg/dose for infants < 1 yr.; 0.045 mg/kg/dose for children ≥ 1 yr.; Maximum dose: 2.3 mg.

VCR (Vincristine) IV on Day 1. 0.025 mg/kg/dose for infants < 1 yr.; 0.05 mg/kg/dose for children 1 yr. to 3 yrs.; 1.5 mg/m2/dose for children >3 yrs.; Maximum dose: 2 mg.

VCR*(Vincristine) IV on Day 1. 0.033 mg/kg/dose for infants < 1 yr.; 0.067 mg/kg/dose for children 1 yr. to 3 yrs.; 2 mg/m2/dose for children > 3 yrs. Maximum dose: 2 mg.

**WT (2) week**

1 2 3 4 5 6**^+^** 7 8 9 10 11 12 13 14 15 16 17 18 19 20 21 22 23 24 25

**DACT DOXO DACT DOXO DACT DOXO* DACT DOXO* DACT**

**VCR VCR VCR VCR VCR VCR VCR VCR VCR VCR VCR* VCR* VCR* VCR* VCR***

**XRT**

**^+^ After surgery/biopsy, some patients may switch to another regimen.**

DOXO (Doxorubicin) IV on Day 1. 1.5 mg/kg/dose for infants < 1 yr.; 45 mg/m2/dose for children >1 yrs.

DOXO* (Doxorubicin) IV on Day 1. 1 mg/kg/dose for infants < 1 yr.; 30 mg/m2/dose for children >1 yrs.

DACT (Dactinomycin) IV on Day 1. 0.023 mg/kg/dose for infants < 1 yr.; 0.045 mg/kg/dose for children ≥ 1 yr.; Maximum dose: 2.3 mg.

VCR (Vincristine) IV on Day 1. 0.025 mg/kg/dose for infants < 1 yr.; 0.05 mg/kg/dose for children 1 yr. to 3 yrs.; 1.5 mg/m2/dose for children >3 yrs.; Maximum dose: 2 mg.

VCR*(Vincristine) IV on Day 1. 0.033 mg/kg/dose for infants < 1 yr.; 0.067 mg/kg/dose for children 1 yr. to 3 yrs.; 2 mg/m2/dose for children > 3 yrs. Maximum dose: 2 mg.

XRT (Radiation therapy) starts within 10 days postoperation for patients whose primary tumors were resected initially.

**WT (3) week**

1 2 3 4 5 6**^+^** 7 8 9 10 11 12 13 14 15 16 17 18 19 20 21 22 23 24 25

**DOXO DOXO DOXO DOXO DOXO**

**VCR VCR VCR VCR VCR VCR VCR VCR VCR VCR VCR* VCR* VCR* VCR***

**CPM CPM* CPM CPM* CPM CPM* CPM CPM***

**ETOP ETOP ETOP ETOP**

**XRT**

**^+^ After surgery/biopsy, some patients may switch to another regimen.**

CPM (Cyclophosphamide) IV on Day 1-5.14.7mg/kg/dose for infants < 1 yr.; 440 mg/m2/dose for children ≥ 1 yr.

CPM* (Cyclophosphamide) IV on Day 1-3.14.7mg/kg/dose for infants < 1 yr.; 440 mg/m2/dose for children ≥ 1 yr.

DOXO (Doxorubicin) IV on Day 1. 1.5 mg/kg/dose for infants < 1 yr.; 45 mg/m2/dose for children >1 yrs.

VCR (Vincristine) IV on Day 1. 0.025 mg/kg/dose for infants < 1 yr.; 0.05 mg/kg/dose for children 1 yr. to 3 yrs.; 1.5 mg/m2/dose for children >3 yrs.; Maximum dose: 2 mg.

VCR*(Vincristine) IV on Day 1. 0.033 mg/kg/dose for infants < 1 yr.; 0.067 mg/kg/dose for children 1 yr. to 3 yrs.; 2 mg/m2/dose for children > 3 yrs. Maximum dose: 2 mg.

ETOP (Etoposide) IV on Days 1-5. 3.3 mg/kg/dose for infants < 1 yr.; 100 mg/m2/dose for children ≥ 1 yr.

XRT (Radiation therapy) starts within 10 days postoperation for patients whose primary tumors were resected initially.

**WT (4) week**

1 2 3 4 5 6**^+^** 7 8 9 10 11 12 13 14 15 16 17 18 19 20 21 22 23 24 25 26 27

**CARBO CARBO CPM CARBO CARBO CPM CARBO CARBO CPM**

**ETOP ETOP DOXO ETOP ETOP DOXO ETOP ETOP DOXO**

**VCR VCR VCR VCR VCR VCR VCR VCR VCR**

**XRT**

**^+^ After surgery/biopsy, some patients may switch to another regimen.**

CARBO (CARBOplatin) IV on Day 1-2. 15mg/kg/dose for infants < 1 yr.; 450 mg/m2/dose for children ≥ 1 yr.

CPM (Cyclophosphamide) IV on Day 1-5.14.7mg/kg/dose for infants < 1 yr.; 440 mg/m2/dose for children ≥ 1 yr.

ETOP (Etoposide) IV on Days 1-5. 3.3 mg/kg/dose for infants < 1 yr.; 100 mg/m2/dose for children ≥ 1 yr.

DOXO (Doxorubicin) IV on Day 1. 1.5 mg/kg/dose for infants < 1 yr.; 45 mg/m2/dose for children >1 yrs.

VCR (Vincristine) IV on Day 1. 0.025 mg/kg/dose for infants < 1 yr.; 0.05 mg/kg/dose for children 1 yr. to 3 yrs.; 1.5 mg/m2/dose for children >3 yrs.; Maximum dose: 2 mg.

XRT ( Radiation therapy ) starts within 10 days postoperation for patients whose primary tumors were resected initially.

**WT (5) week**

1 2 3 4 5 6^+^

IFO VCR IFO VCR

ETOP ETOP

VCR VCR

^+^: Reassessed for feasibility of surgical management.

IFO (Ifosfamide) IV on Day 1-5. 1.5g/m2/dose for children ≥ 1 yr. 75% dose for infants < 1 yr.; 50% dose for infants < 6 months.

ETOP (Etoposide) IV on Days 1-5. 3.3 mg/kg/dose for infants < 1 yr.; 100 mg/m2/dose for children ≥ 1 yr.

VCR (Vincristine) IV On Day 1. 0.025 mg/kg/dose for infants < 1 yr.; 0.05 mg/kg/dose for children 1 yr. to 3 yrs.; 1.5 mg/m2/dose for children >3 yrs.; Maximum dose: 2 mg.
